# Genetic analysis and clinical features of X-linked retinoschisis in Chinese patients

**DOI:** 10.1038/srep44060

**Published:** 2017-03-08

**Authors:** Qin-rui Hu, Lv-zhen Huang, Xiao-li Chen, Hui-ka Xia, Tian-qi Li, Xiao-xin Li

**Affiliations:** 1 Department of Ophthalmology, Peking University People’s Hospital, Key Laboratory of Vision Loss and Restoration, Ministry of Education, Beijing Key Laboratory for the Diagnosis and Treatment of Retinal and Choroid Diseases, Xizhimen South Street 11, Beijing, China.

## Abstract

Many mutations in the retinoschisis (RS1) gene have been identified, but there are limited clinical data relating to the different genotypes. This study investigated the genotype, clinical phenotype and therapies for X-linked juvenile retinoschisis (XLRS) patients in China to evaluate the effects of gene mutations and therapies on the prognosis of the disease. Thirty patients were recruited in the study. Genetic examination identified 8 novel RS1 gene mutations. Twenty-four patients were identified as missense mutation, which was the most common gene mutation in XLRS patients. Amino acids 102 and 209 were the most common mutation areas, accounting for a total 35.7% of all patients. Mutations affecting amino acid 102 were associated with poor results on the flash electroretinogram (ERG). Sixteen patients had various complications. Anti-vascular endothelial growth factor (VEGF) drugs were given to four patients with hemorrhage or other complications, and serious adverse events did not occur. Our outcome demonstrates that missense mutation was the leading cause of XLRS and more than half of the patients with this missense had various complications. Anti-VEGF drugs may be an effective and safe way to prevent deterioration of XLRS with certain complications. There is wide genotypic and phenotypic variability in Chinese patients with XLRS.

X-linked retinoschisis (XLRS) is an inherited vitreoretinal dystrophy characterized by foveal schisis within the inner retinal layers in young patients^1^,^2^,^3^. The estimated prevalence of XLRS ranges from 1:5000 to 1:25000^3^,^4^,^5^. Retinoschisis, first described by Hass in 1898, has the clinical feature of early visual loss associated with the bilateral foveae and retinoschisis is the most common clinical finding in young patients^6^,^7^. The RS1 gene has been identified as a noteworthy disease-causing gene by Sauer CG *et al*.^8^. Its entire coding region may be readily sequenced, since the gene contains only six exons and produces a transcript under 700 base pairs in size. Since the gene was first characterized, a large number of relevant studies have contributed to improving the diagnosis and understanding of the disease.

However, XLRS is characterized by a high degree of clinical variability in male children^4^. There is currently no appropriate approach to halt the progressive maculopathy in XLRS, so the clinical management is mainly for the therapy of complications^7^.

Appropriate therapies to achieve a better prognosis for XLRS have been pursued for decades. Here, we reviewed the molecular genetics, clinical features, and therapeutic options of XLRS patients in our department, both to improve the clinical management and to better understand the retinal function and development of XLRS.

## Results

### Molecular diagnosis

**Figure 1:**
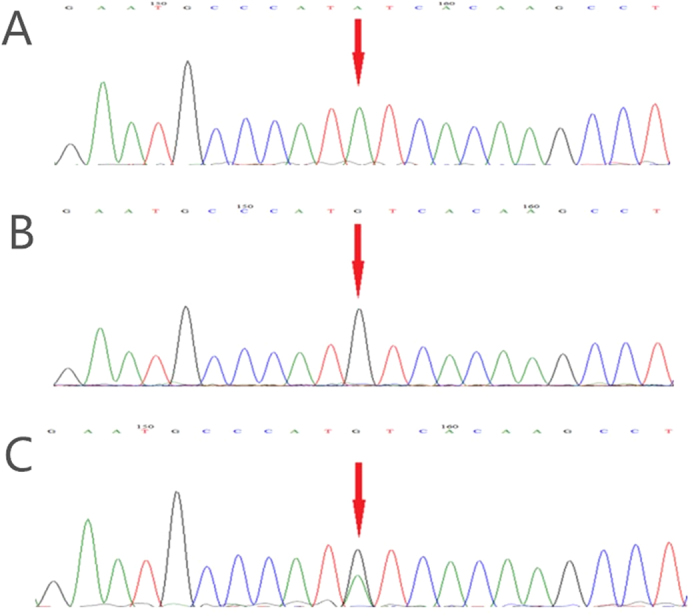
Genetic tests of one patient and his parents. (**A**) A nucleotide mutation was found in the RS1 gene on the X chromosome in one male patient (RS1, chrx: 18665443, c.194A > G, i.e., nucleotide 194 of the coding sequence changed from A to G). The mutation results in a change of amino acid 65 from Tyr to Cys (p.Tyr65Cys). This mutation is classified as a missense mutation. (**B**) His father did not show an unusual genotype at the locus. (**C**) His mother was heterozygous at the locus.

Thirty suspected patients, 51 parents and 100 control subjects underwent genetic tests. In total, 28 mutations were identified, 8 of which (28.6%) were novel. There were 24 (85.7%) missense mutations of the RS1 gene, 2 splicing mutations, 1 deletion mutation, and 1 nonsense mutation (Table 1). Two of the 30 patients were not found to have any RS1 gene mutation. The most common mutation occurred in the coding region, specifically at nucleotide 305 of exon 4 (14.3%). These mutations brought about various amino acid changes. Amino acids 102 and 209 were the most common sites of variation, each accounting for 17.9% (5/28) of the predicted amino acid changes. The pathogenic genes were proved to be inherited from the maternal side (Fig. 1). Eight patients reported family histories covering their relatives within three generations.

### Clinical manifestations

**Figure 2:**
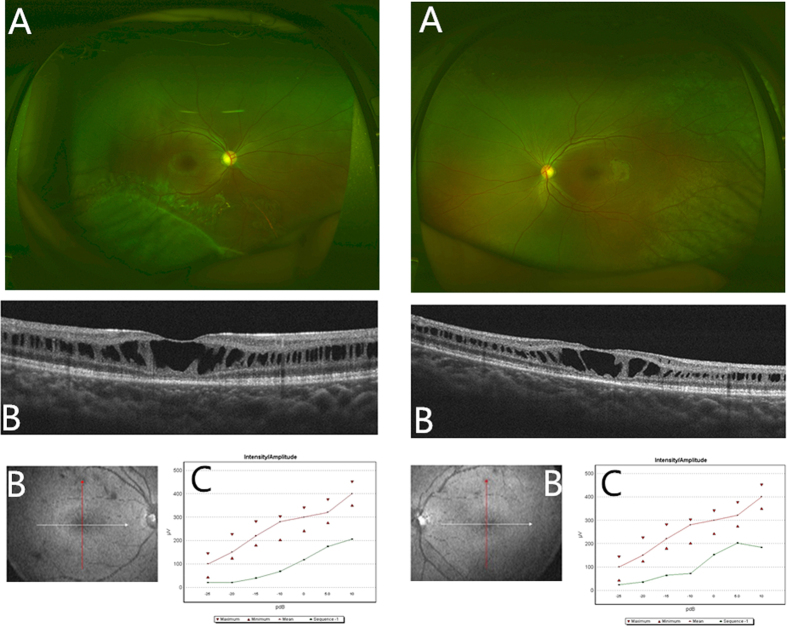
The images of one X-linked retinoschisis patient by various examinations. (**A**)Wide-angle laser fundus photographs showed peripheral retinal splitting in the right eye and macular fold in the left eye. (**B**) Optical coherence tomography images showed the typical retinoschisis of the retinal nerve fiber layer. (**C**) The flash electroretinogram (ERG) displayed the decreased change in the b-waves of both eyes under different light stimulations. The red line represented the mean reference value of the b-wave by ERG test in normal controls in our hospital. The green line represented the measured value of the b-wave for the patient.

The clinical characteristics are summarized in Table 2. Data were collected from 2004 to 2016. Twenty-five patients were included, and 5 patients (patients13, 26, 27, 28, 30) were excluded as their clinical dates were lost or without XLRS mutations. The median age at the time of examination was 7 years, with a range from 7 months to 38 years. The mean visual acuity (VA) was 0.77 ± 0.41. Among the 25 patients, macular (foveal) retinoschisis was present in 22 eyes (22/50, 44.0%), peripheral retinoschisis in 19 eyes (38.0%, mainly in temporal or inferior), total retinal detachment in 10 eyes (20.0%), macular scar in 2 eyes (4.0%) and unknown in 1 eye (2.0%) that had a severe cataract. Figure 2 shows typical XLRS images of 1 patient.

### Electrophysiology

Flash-ERG was performed on 28 eyes of 14 patients (Table 3). ERG demonstrated reduced b-wave amplitudes with relative preservation of a-wave amplitudes in 14 eyes (50.0%). Both a- and b-wave amplitudes were reduced in 12 eyes (42.9%); 2 eyes (7.1%) had relatively normal a- and b-wave amplitudes. The b/a ratio was reduced (<1.2) in 24 eyes (85.7%).

### Complications and therapies

Sixteen patients (16/25, 64.0%) had various complications: 6 patients (10 eyes) had retinal detachments, 7 patients (8 eyes) had vitreous hemorrhages, 6 patients had strabismus and 3 patients (3 eyes) had cataracts. 14 patients (87.5%) in this group belonged to missense mutation. Out of 7 patients with known novel mutations and clinical data, only 2 patients (patients 15, 29) had no complications observed. Among these, 9 patients (36.0%), with an average VA of 0.98 ± 0.42, underwent surgery. The surgeries included intravitreal injection of ranibizumab (IVR) for vitreous hemorrhage in 3 eyes, IVR for exudative retinal detachment in 2 eyes, scleral buckling and cryotherapy for retinal detachment in 4 eyes, intravitreal injection of ganciclovir (IVG) for exudative retinal detachment in 2 eyes and intravitreal injection of bevacizumab (IVB) for vitreous hemorrhage in 1 eye. Vitrectomy, cataract surgery and laser therapy were performed in 1 eye each (Table 2). The average VA after surgery was 0.87 ± 0.38. There was no significant difference in the VA change between preoperative and postoperative tests (P < 0.18) with a mean follow-up visit of 19.7 ± 13.3 months.

## Discussion

Our results confirm that RS1 gene mutation is the primary factor in the development of XLRS. XLRS patients possess the characteristic of high incidence of various complications. Anti-vascular endothelial growth factor (anti-VEGF) drugs may be valid to control hemorrhage and other complications in XLRS. There is wide genotypic and phenotypic variability in Chinese patients with XLRS.

More than one hundred RS1 mutations have been identified (http://www.dmd.nl/rs). In our study, missense mutation was the leading cause of XLRS, with a proportion of 85.7% in all the mutations for the patients, which is similar to previous reports^7^. Interestingly, a large proportion of mutations (28.6%) were novel, which suggests that the mutations may occur at different frequencies in different ethnic groups. The novel mutations included Arg102Cys, Gly91Cys, Arg102Pro, Tyr93Cys, Arg209Cys, splicing mutations (675 + 14C > T), p.95 deletion (c.282_284del), and a nonsense mutation (Ser73Ter). According to our previous study, severe XLRS phenotypes are associated with Arg102Gln, Asp145His, Arg209His, and Arg213Gln mutations^9^. In this investigation, we found that Arg102Gln and Arg209His together accounted for a considerable proportion (35.7%) of mutations. High incidence was associated with these specific mutations for XLRS patients in our study. However, patients with same genetic mutations did not always have the same degree of pathological changes in our study. We presumed that other genetic influences (i.e., environmental factors), might also contribute to disease severity^7^. Sporadic XLRS cases and novel mutations have been reported in China. Recently, two novel mutations were found in a cohort of six unrelated Chinese families^10^. In a similar study, six different RS1 mutations were identified in Chinese families with a clinical diagnosis of XLRS^11^. In that article, a new splice site mutation was described. Yangyan Xiao *et al*. investigated a Chinese family with XLRS and identified a novel mutation in the RS1 gene^12^. The mutation co-segregates with disease in the family and likely results in loss of RS1 protein production. The mutation expands the mutational spectrum of XLRS, and the phenotypes of the affected members of this family also provide evidence of potential genetic or environmental factors on XLRS prognosis. A study screened RS1 mutations from 16 patients belonging to 16 Chinese families^13^. In that study, sixteen different mutations were identified, nine of which had not been previously reported. The investigators concluded that no obvious genotype-phenotype association was observed in their Chinese patients. Our previous study identified severe XLRS phenotypes associated with some frameshift mutations, splice donor site mutations, and missense mutations^9^. We described the wide variability in the phenotype in Chinese patients with XLRS and expanded the information on clinical manifestations. Undoubtedly, the work described above will facilitate the diagnosis, appropriate early therapy, and genetic counseling and therapy based on the prognosis of XLRS.

Eyes with typical ERG manifestation (relatively normal a-wave and reduced b-wave amplitudes) accounted for 50.0% of all those that underwent ERG examinations in our study. The result was similar to those of previous studies^7^,^14^,^15^,^16^. Eyes with reductions in both a- and b-wave amplitudes composed a large proportion (42.9%) of the sample. A decreased response may be related to serious retinoschisis and complications including vitreous hemorrhage and cataracts. It is noted that specific mutation may have severity ERG results. In our study, patients with amino acid 102 mutations (patients 1, 2, 4, 21) had lower a- and b-wave amplitudes compared with other mutations. The measurements were consistent with their clinical manifestations. ERG is an important approach contributing to differential diagnosis. One patient in our study was suspected to have indistinct retinal detachment initially. However, it proved to be merely retinoschisis, as ERG test showed that the suspected eye had relatively normal a- and b-waves compared with the other eye without detachment. In the era of gene therapy, ERG is becoming an important indicator of the effects of therapy for both humans and animal models. Our study provides a large amount of clinical data for the utility of ERG. Many animal experiments use ERG as an important reference standard of eye function; however, many patients in the clinic have normal ERG manifestations, as we have mentioned. For these patients, we need to seek a more comprehensive measurement instead to evaluate the effects of therapy.

**Figure 3:**
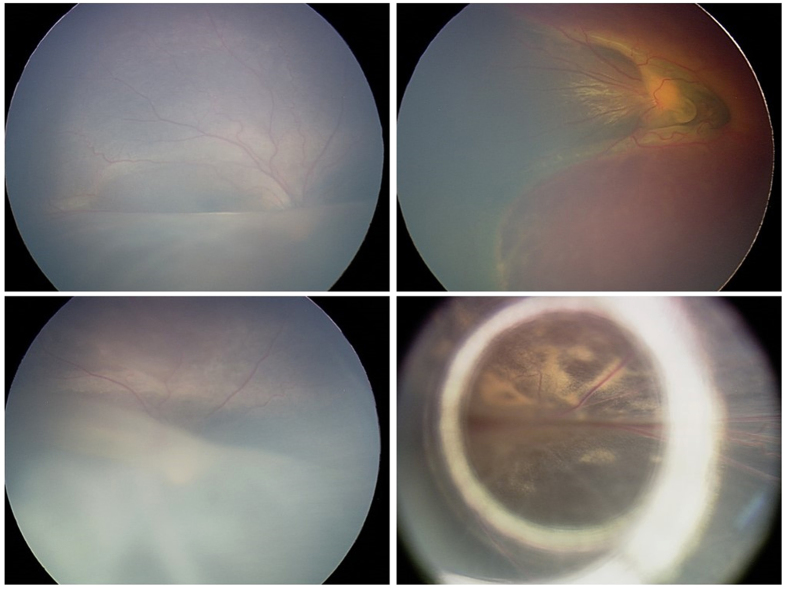
Fundus images of patient 2. The patient had cytomegalovirus (CMV) infection, retinopathy of prematurity (ROP) and exudative retinal detachment in eyes. Both eyes underwent intravitreal injection of ranibizumab 5 times. (**A**) Fundus image of the right eye showed exudative retinal detachment and tortuous vessel. (**B**) Fundus image of the left eye showed the hemorrhage around the optic disc. (**C**) Fundus image of the right eye showed the vessel turned normal and the inferior retina became rigid after the treatment. (**D**) Fundus image of the left eye showed the hemorrhage disappeared while white-yellow areas of the fundus appeared after the treatment.

**Figure 4:**
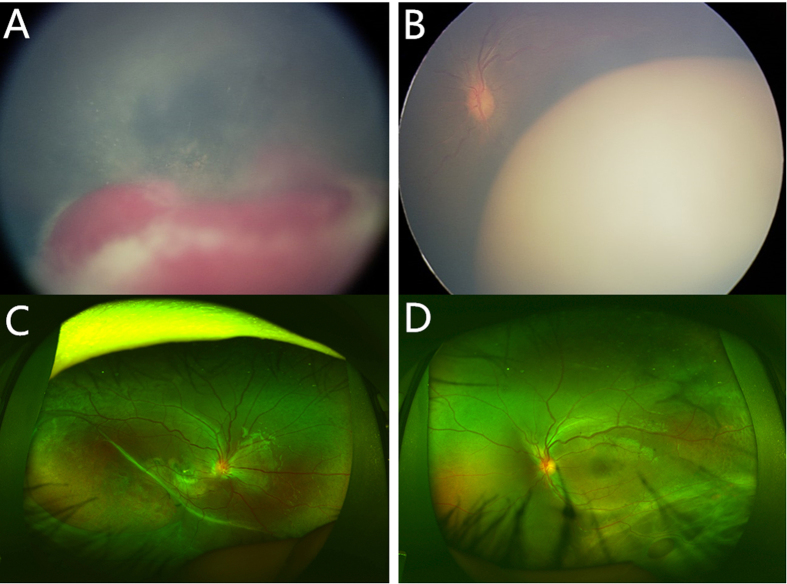
Fundus images of patient 9. Hemorrhage occurred in the right and left eye in succession within half a year in patient 9. (**A**,**B**) Vitreous hemorrhage opacity was found in the right eye and tortuous vessel was found in the left eye. Both eyes received anti-VEGF drugs treatment twice. (**C**,**D**) The images showed wide-angle laser fundus photography of both eyes two years after the last therapy. The peripheral splitting areas were stable and the rest area of the fundus turned relatively normal in both eyes.

In our study, 64.0% of the patients had various complications, and 1/3 of them required surgical intervention. Out of 16 cases with complications, 14 cases belonged to missense mutation. Patients with missense mutation tend to have severe complications and require surgery. The retinoschisis that occurs within the inner retina in XLRS leads to the decrease in vision in the children, but there used to be no effective therapy^7^,^17^. Scleral buckling for retinal detachment was performed in 4 eyes with rhegmatogenous retinal detachment as a traditional method^18^. In our patients, abnormal vasculature with hemorrhage was not rare; this condition can cause surface wrinkling or traction macular detachment due to the abnormal vascular proliferation and leakage^19^,^20^. In this study, three patients were treated a total of 7 times with anti-VEGF drugs. Patient 2 (Fig. 3), who had typical retinopathy of prematurity (ROP) and cytomegalovirus (CMV) infection, was treated 5 times with binocular IVR, requiring more repeat dosing than simple ROP^21^. Furthermore, in a study by Spaide RF *et al*., intravitreal injection of bevacizumab in patients with proliferative diabetic retinopathy complicated by vitreous hemorrhage resulted in rapid resolution of vitreous hemorrhage^22^. In our study, three patients (patients 1, 9, 16) received IVR and/or IVB treatment for hemorrhages by an average of 1.8 times each eye. Remarkably, the hemorrhages reduced rapidly, and few complications were found after the treatment (Fig. 4). This group achieved relatively preserved VA at follow-up visits more than one year later. The results support the above conclusion and suggest that anti-VEGF drugs may be an effective way to control hemorrhage and other complications in XLRS. It is also interesting to notice that patient 25, who had severe exudative retinal detachment (CMV infection), underwent scleral buckling and cryotherapy in the left eye for proliferative retinopathy after binocular transscleral drainage of subretinal fluid initially. After that, the subretinal fluid relapsed in both eyes. The aqueous humor tested negative for CMV-IgG by polymerase chain reaction (PCR), but the subretinal fluid tested positive. The disease was eventually improved and stabilized by 4 treatments with binocular IVG. Systemic adverse reactions did not appear during 6 years of follow-up. This case indicates that CMV infection can affect the posterior segment of the eye while leaving the anterior segment normal and that aqueous humor testing is not a reliable basis for a final diagnosis. XLRS could be controlled by IVR or IVG without serious adverse events. Timely and effective measures for complications are required.

Genetic testing, combining with optical coherence tomography (OCT) and ERG, is the most definitive evidence for diagnosis of XLRS. However, two patients in our study had none RS1gene mutations. To the best of our knowledge, it is not easy to distinguish atypical XLRS from familial exudative vitreoretinopathy (FEVR), rod-cone degeneration disease and other diseases. In a clinical study by Dhananjay Shukla, severe dragging of the macula was the most striking clinical finding in five XLRS patients^23^. Extensive mottling and atrophy of the retinal pigment epithelium at the posterior pole was presented in three XLRS patients. Differential diagnosis for such disease includes both infectious and noninfectious conditions like ROP, FEVR, toxocariasis, toxoplasmosis, tuberculosis, Coats disease, etc. Therefore, using varies kinds of tests skillfully and being familiar with the clinical features of various diseases for clinician will help to avoid misdirected investigations and interventions for mimicking diseases.

Genead MA found that patients with XLRS had the potential to experience a beneficial effect from sustained treatment with 2% dorzolamide, as significant improvement in the foveal zone thickness was observed^24^. The result suggests that a mechanism independent of retinoschisin itself is responsible for the improvement in the degree of cystic spaces^25^. However, the case series by Khandhadia S found that this did not necessarily correlate with improvement in VA and that genotypic information did not predict the response to this treatment^26^. Nevertheless, it might have a role to play in combination with gene or surgical therapies by quickly reducing cavity size, which would aid the healing process by bringing tissue together and by setting the stage for inducing RS protein expression by gene transfer to stabilize the retina over a longer term^27^.

Several mouse models of XLRS have been created, mainly by genetic engineering, and remarkable progress has been achieved recently. Yong Zeng *et al*. created an XLRS mouse model (RS1-KO) by substituting a neomycin resistance cassette for exon 1 and 1.6 kb of intron 1 of Rs1h, the murine orthologue of the human RS-1 gene^28^. The mouse mimics structural features of human XLRS with dissection through, and disorganization of, multiple retinal layers and implicates a synaptic transmission deficit. Replacement therapy by supplementing normal Rs1h protein induced retinoschisin expression and restored the normal ERG configuration. Another study demonstrated that restoration of RS1 via retina-specific delivery of adeno-associated virus type 8-RS1 (AAV8-RS1) vector rescued molecular pathology and restored function in adult Rs1h-KO animals^29^. Abu E. Bashar delivered syngeneic adipose-derived mesenchymal stem cells (MSCs) that were genetically modified to secrete human RS1 to the retina of the Rs1h knockout mouse model by intravitreal injection^30^. RS1-expressing MSCs were found mainly in the inner retinal layers. Four months later, the schisis cavity areas were reduced by 78%, more photoreceptor nuclei were present, and the ERG b-wave was significantly improved after multiple injections. Studies in rabbits and Rs1h-KO mice of a recombinant AAV8 vector for X-linked retinoschisis gene therapy showed retinal detachment without other ocular or systemic adverse events^31^. Although biodistribution analysis detected vector genome in extraocular tissues of Rs1h-KO mice, no evidence of damage was found in organs or tissues. On this basis, a human trial was carried out recently with the AAV8-RS1 vector to probe synaptic plasticity in XLRS patients^29^.

Safe and effective gene therapy in an animal model eventually provides a basis for gene-directed therapy. However, it is unknown how the mutant protein would interact with normal retinoschisin protein or at what level it would be expressed inside the cell. Studies are under way to gain insight into this. As we know, gene therapy could not deal with the patients with serious complications mentioned above. Restore the relative normal structure before the gene therapy may be a preferred option to cure this disease. Our study provided abundant clinical and genetic data to the comprehensive treatment with gene therapy. However, we still need to address the possible challenges of gene therapy, including the timing of gene intervention, immune response control, complication management, interaction with other treatments and ocular and systemic adverse events in the long run.

In conclusion, RS1 gene mutation is a major cause of XLRS. OCT and ERG tests typically feature in the diagnostic process. Molecular genetic examination confers advantages in diagnosis and differential diagnosis. XLRS complications are variable in clinical practice. Timely and targeted measures should be considered when managing the complications to prevent progressive visual deterioration and improve visual prognosis.

## Methods

The research protocol was approved by the ethics review board of the Peking University School of Medicine. The study procedures were carried out in accordance with institutional guidelines and the Declaration of Helsinki. Informed consent was obtained from all patients after a full explanation of the procedures.

Thirty suspected male patients were enrolled in this study from 2004 to 2016. Patients underwent a comprehensive ophthalmological examination including slit-lamp biomicroscopy, funduscopic examinations, measurements of best corrected visual acuity (VA) with a logarithmic VA chart, OCT examinations (Spectralis; Heidelberg Engineering, Heidelberg, Germany) and ERG tests (RETIport, ROLAND CONSULT, Germany). Clinical therapies were recorded during the follow-up visit. Molecular genetic examinations of the RS1 gene were performed in the 30 males, 51 parents, and 100 control subjects (normal vision and without any eye diseases). Genomic DNA was extracted using an Agilent SureSelect Target Enrichment System Kit (Agilent, USA). Polymerase chain reaction (PCR) was performed to amplify six exons of the RS1 gene. Samples were sequenced directly by loading the sequencing reaction product onto an NEXTSEQ500 (Illumina, USA). T test was carried out on the process of clinical data comparison (SPSS 16.0). P value of 0.05 was considered to be statistically significant.

## Additional Information

**How to cite this article:** Hu, Q.-r. *et al*. Genetic analysis and clinical features of X-linked retinoschisis in Chinese patients. *Sci. Rep.* **7**, 44060; doi: 10.1038/srep44060 (2017).

**Publisher's note:** Springer Nature remains neutral with regard to jurisdictional claims in published maps and institutional affiliations.

## Figures and Tables

**Table 1:** RS1 genetic testing results.

Patients	Mutations	Predicted amino acid changes	Mutation types	Reported before (Y/N)	Parents	Family history (Y/N)
1	c.305G > A	p.Arg102Gln	missense	Y	Mother heterozygote	Y
2	c.421C > T	p.Arg102Cys	missense	N	Mother heterozygote	N
3	c.271G > T	p.Gly91Cys	missense	N	Mother heterozygote	Y
4	c.305G > C	p.Arg102Pro	missense	N	Mother heterozygote	N
5	c.214G > A	p.Glu72Lys	missense	Y	Mother heterozygote	Y
6	c.326G > A	p.Gly109Glu	missense	Y	Mother heterozygote	N
7	c.675 + 14C > T	—	splicing	N	Mother heterozygote	N
8	c.626G > A	p.Arg209His	missense	Y	Mother heterozygote	Y
9	c.194A > G	p.Tyr65Cys	missense	Y	Mother heterozygote	N
10	c.625C > T	p.Arg209Cys	missense	Y	Mother heterozygote	N
11	c.626G > A	p.Arg209His	missense	Y	Mother heterozygote	N
12	c.282–284del	p.95del	delete	N	Mother heterozygote	Y
13	none	—	—	—	—	N
14	c.276G > T	p.Trp92Cys	missense	Y	Mother heterozygote	N
15	c.278A > G	p.Tyr93Cys	missense	N	Mother heterozygote	N
16	c.216G > T	p.Glu72Asp	missense	Y	Mother heterozygote	N
17	c.421C > T	p.Arg141Gly	missense	Y	Mother heterozygote	N
18	c.637C > T	p.Arg213Trp	missense	Y	Mother heterozygote	N
19	c.625C > T	p.Arg209Cys	missense	Y	Mother heterozygote	N
20	c.544C > T	p.Arg182Cys	missense	Y	Mother heterozygote	Y
21	c.305G > A	p.Arg102Gln	missense	Y	Mother heterozygote	N
22	c.214G > A	p.Glu72Lys	missense	Y	Mother heterozygote	Y
23	c.305G > A	p.Arg102Gln	missense	Y	Mother heterozygote	N
24	c.221G > T	p.Gly74Val	missense	Y	Mother heterozygote	Y
25	c.589C > T	p.Glu72Asp	missense	Y	Mother heterozygote	N
26	c.216G > T	p.Arg209Cys	missense	N	Mother heterozygote	N
27	c.523–2A > G	—	splicing	Y	Mother heterozygote	N
28	c.418G > A	p.Gly140Arg	missense	Y	Mother heterozygote	N
29	c.218delC	p.Ser73Ter	nonsense	N	Mother heterozygote	N
30	none	—	—	—	—	N

Blank sections mean no data available.

**Table 2:** Clinical data of X-linked retinoschisis patients.

Patients	Gender	Age (years)	Complications	Surgeries	VA (first visit)	VA (after surgery)		
					OD	OS	OD	OS
1	Male	7	OS vitreous hemorrhage OD macular epiretinal membrane	OS IVR*1	LP	0.4	LP	0.4
2	Male	10	OS vitreous hemorrhage/cataract OU exudative retinal detachment/CMV/ROP	OS P + I OU IVR*5	1.5	1.0	1.0	1.6
3	Male	3	OS cataract OD glaucoma OU RRD	—	NLP	NLP	—	—
4	Male	10	OS RRD OU retinal dragging	OS Scleral buckling + cryotherapy	1.2	0.8	1.2	0.7
5	Male	4	—	—	0.7	0.7	—	—
6	Male	6	OD vitreous hemorrhage	OD Laser	0.7	0.7	0.6	0.5
7	Male	8	OS RRD	OS scleral buckling + cryotherapy	0.9	1.6	0.7	1.3
8	Male	3	strabismus	—	—	—	—	—
9	Male	6	OU vitreous hemorrhage	OS IVR*2 OD IVR*1/IVB*1	1.3	1.1	1.1	1.1
10	Male	6	—	—	0.4	0.4	—	—
11	Male	5	strabismus	—	0.8	1.4	—	—
12	Male	38	OS cataract/glaucoma OD vitreous hemorrhage/trauma	—	NLP	0.4	—	—
14	Male	11	—	—	0.2	0.2	—	—
15	Male	3	—	—	0.6	0.6	—	—
16	Male	7	OS vitreous hemorrhage OU RD	OS IVB*2 OU scleral buckling	LP	0.7	LP	0.7
17	Male	5	—	—	—	—	—	—
18	Male	25	strabismus	—	LP	0.4	—	—
19	Male	11	—	—	—	—	—	—
20	Male	3	strabismus	—	—	—	—	—
21	Male	8	—	—	0.1	1.0	—	—
22	Male	7	—	—	0.6	0.6	—	—
23	Male	8	OS vitreous hemorrhage	OS vitrectomy	1.0	0.4	1.0	0.3
24	Male	10	strabismus	—	0.3	1.3	—	—
25	Male	0.6	OU exudative retinal detachment/CMV strabismus	OD IVG*3 OS scleral buckling + cryotherapy/IVG*1 OU transscleral drainage	1.5^a^	0.8^a^	1.0	0.6
26	Male	defect	defect	defect	defect	defect	—	—
27	Male	defect	defect	defect	defect	defect	—	—
28	Male	defect	defect	defect	defect	defect	—	—
29	Male	10	—	—	0.3	0.3	—	—

A: grating acuity. CMV: cytomegalovirus infection. IVR: intravitreal injection of ranibizumab. IVG: intravitreal injection of ganciclovir. LP: light perception. NLP: no light perception. OS: oculus sinister, left eye. OD: oculus dexter, right eye. OU: oculus uterque, binoculus. P + I: phacoemulsification + intraocular lens implantation. RD: retinal detachment. VA: visual acuity. Asterisks means times of therapy. Blank sections mean no data available.

**Table 3:** Flash electroretinogram data of X-linked retinoschisis patients (scotopic, white flash).

Patients	a-wave (μv)	b-wave (μv)	b/a
1	295	258	0.9
	89	76	0.9
2	153	117	0.8
	170	130	0.8
4	227	173	0.8
	66.5	74.5	1.1
5	84	98	1.2
	128	180	1.4
6	319	204	0.6
	385	255	0.7
7	52.1	35.4	0.7
	20.2	38.1	1.9
9	513	316	0.6
	506	308	0.6
10	326	324	1.0
	355	361	1.0
13	209	119	0.6
	424	216	0.5
18	73.4	47.4	0.6
	213	77.8	0.4
21	124	60.4	0.5
	189	70.1	0.4
22	521	443	0.9
	489	382	0.8
23	173	199	1.2
	326	338	1.0
24	367	208	0.6
	311	189	0.6
	197 (58)*	354 (103)*	1.8 (0.6)*

Asterisks indicate mean values (2 standard deviations [SD]) in our lab.
